# Gut Dysbiosis and Immune System in Atherosclerotic Cardiovascular Disease (ACVD)

**DOI:** 10.3390/microorganisms10010108

**Published:** 2022-01-05

**Authors:** Ji Youn Yoo, Sarah Sniffen, Kyle Craig McGill Percy, Veera Bramhachari Pallaval, Bojjibabu Chidipi

**Affiliations:** 1 College of Nursing, University of Tennessee, 1200 Volunteer Blvd, Knoxville, TN 37996, USA; 2 Department of Neuroscience, College of Medicine, University of Florida, Gainesville, FL 32610, USA; sarah.sniffen@ufl.edu; 3 Department of Pathology, School of Medicine, Case Western Reserve University, Cleveland, OH 44106, USA; kcm78@case.edu; 4 Department of Biotechnology, Krishna University, Machilipatnam 521004, Andhra Pradesh, India; veerabramha@gmail.com; 5 Morsani College of Medicine, University of South Florida, 12901 Bruce B. Downs Blvd., MDC 78, Tampa, FL 33612, USA; chidipib@usf.edu

**Keywords:** atherosclerotic cardiovascular disease (ACVD), atherosclerosis, gut dysbiosis, immune system, gut microbial metabolites, SCFAs, TMAO

## Abstract

Atherosclerosis is a leading cause of cardiovascular disease and mortality worldwide. Alterations in the gut microbiota composition, known as gut dysbiosis, have been shown to contribute to atherosclerotic cardiovascular disease (ACVD) development through several pathways. Disruptions in gut homeostasis are associated with activation of immune processes and systemic inflammation. The gut microbiota produces several metabolic products, such as trimethylamine (TMA), which is used to produce the proatherogenic metabolite trimethylamine-N-oxide (TMAO). Short-chain fatty acids (SCFAs), including acetate, butyrate, and propionate, and certain bile acids (BAs) produced by the gut microbiota lead to inflammation resolution and decrease atherogenesis. Chronic low-grade inflammation is associated with common risk factors for atherosclerosis, including metabolic syndrome, type 2 diabetes mellitus (T2DM), and obesity. Novel strategies for reducing ACVD include the use of nutraceuticals such as resveratrol, modification of glucagon-like peptide 1 (GLP-1) levels, supplementation with probiotics, and administration of prebiotic SCFAs and BAs. Investigation into the relationship between the gut microbiota, and its metabolites, and the host immune system could reveal promising insights into ACVD development, prognostic factors, and treatments.

## 1. Introduction

Atherosclerosis is a condition characterized by plaque build-up within the arteries and thickening of the arterial walls. It is widely regarded as the leading cause of cardiovascular disease (henceforth referred to as atherosclerotic cardiovascular disease (ACVD)), which is currently the primary cause of death worldwide [1]. Obesity, diabetes, and metabolic syndrome are the most common factors contributing to an increased risk of developing cardiovascular disease. Together with atherosclerosis, these comorbidities can contribute to an increased risk of myocardial infarction, cerebral infarction, and other cardiovascular diseases [2]. Nevertheless, the origin and pathogenesis of atherosclerosis remain unclear.

Over the last 20 years, numerous studies have emphasized the role inflammation plays in ACVD. Cholesterol deposition, inflammation, extracellular matrix formation, and the gut microbiota elevate the risk of ACVD development. Understanding how the gut microbiota modulates risk factors for ACVD could yield predictive and prognostic data of extensive clinical utility [3,4,5]. The gut microbiota acts as a primary regulator of physiology and function, and as a requisite for immune system development and regulation, and for maintaining intestinal homeostasis [6,7]. A healthy gut principally comprises the commensal bacterial phyla *Actinobacteria*, *Bacteroidetes*, *Firmicutes*, and *Proteobacteria*, with *Firmicutes* and *Bacteroidetes* being the most prevalent. These enteric bacteria break down proteins and polysaccharides, synthesize vitamins, and generate a wide variety of metabolic products. These include trimethylamine (TMA), which is converted to trimethylamine N-oxide (TMAO), short-chain fatty acids (SCFAs), and bile acids (BAs). These metabolites are involved in regulating major host metabolic and immune pathways including interactions with immune cells such as dendritic cells (DCs), macrophages, and T cells [8]. However, when pathogenic bacteria dominate, as in gut dysbiosis conditions, these beneficial metabolic processes are disrupted. Therefore, manipulating the gut microbiota and its host interactions has great potential for maintaining gut homeostasis, directly resulting in improved ACVD outcomes.

This review provides a synopsis of the experimental and clinical evidence for the crucial role the gut microbiota plays in ACVD. We assess how alterations in the gut microbiota composition and gut metabolism lead to changes in the regulation of the immune system, resulting in the propagation of ACVD. Finally, this review emphasizes how new insights into the gut microbiota–ACVD relationship may lead to the expansion of therapeutic strategies for ACVD.

## 2. Gut Dysbiosis in ACVD

The resident bacteria of the gut can activate a wide variety of host activities. Dysbiosis of the gut is the common denominator in many risk factors for ACVD including lifestyle, dietary habits, aging, and obesity [9]. Recently, substantial interest has centered around how changing the gut microbiota composition can lead to changes in the host metabolism, affecting ACVD pathogenesis. Depending on the metabolites present, pro- or anti-inflammatory processes are activated [9]. Therefore, atherosclerosis pathophysiology is made up of both metabolic and inflammatory aspects, which can both be affected by alterations in the gut microbiota composition.

The gut microbial community is largely composed of four phyla including *Actinobacteria*, *Bacteroidetes*, *Firmicutes*, and *Proteobacteria* [10]. In a healthy gut, the anaerobic bacteria *Firmicutes* and *Bacteroidetes* constitute over 90% of the bacteria present in the gut, but the proportion of *Firmicutes* to *Bacteroidetes* differs across individuals [11,12]. This interindividual variation in bacterial composition can be attributed to differences in the host genome and environmental factors, specifically stress levels, the use of antibiotics, and diet [10]. Maintaining immune homeostasis is a highly demanding task, requiring differentiation between the multitude of beneficial microbes and the scant opportunistic pathogens. Gut homeostasis is achieved when the obligate anaerobic *Firmicutes* and *Bifidobacteriaceae*, which is a family of *Actinobacteria*, dominate, whereas proliferation of facultative anaerobic *Enterobacteriaceae*, which is a family of *Proteobacteria*, generally indicates gut dysbiosis [13].

Over time, it is becoming clearer that the interactions between the gut microbiota and the immune system lead to ACVD. Haraszthy et al. showed that atherosclerotic plaques in human endarterectomy specimens contain various species of bacterial DNA such as *Prevotella intermedia*, *Porphyromonas gingivalis*, *Actinobacillus actinomycetem comitans*, and *Bacteroides forsythus*. Additionally, they found that periodontal pathogens and infectious microorganisms, such as *Chlamydia pneumoniae*, may contribute to the pathogenesis of atherosclerosis, leading to ACVD [14]. Ziganshina and colleagues (2016) found that the order *Burkholderiales* and the genus *Curvibacter* were elevated in patients’ atherosclerotic plaque samples [15]. Voronina et al. showed that *Burkholderiales* and other members of *Ralstonia* and *Propionibacterium* represented the most elevated taxa within atherosclerotic plaques [16]. Some bacteria found in plaques were also associated with several clinical traits, such as alanine aminotransferase, total cholesterol, and fibrinogen levels [17]. Karlsson and colleagues (2012) demonstrated that gut microbiota, especially *Collinsella*, were abundant in symptomatic atherosclerosis patients [18]. *Collinsella* has been identified to affect the host metabolism via decreasing glycogenesis in the liver, changing cholesterol absorption in the gut, and increasing triglyceride synthesis [17]. Another important function of *Collinsella* was its ability to decrease the expression of tight junction proteins, leading to increased gut permeability [19]. Furthermore, *Collinsella* impacts the epithelial production of Interleukin 17A(IL-17A), C-X-C Motif Chemokine Ligand 1 (CXCL1), and C-X-C Motif Chemokine Ligand 5 (CXCL5) [19]. This may lead to neutrophil recruitment and nuclear factor kappa-light-chain-enhancer of activated B cells (NF-kB) activation, both of which contribute to the pathologic effects of disease-causing organisms in the gut [19]. Liu et al. (2020) compared gut bacterial diversity in Apolipoprotein E knockout (ApoE^−/−^) mice vs. wild-type mice both fed high-fat diets. The group found that as atherosclerosis developed in the ApoE null mice, their gut bacterial composition changed. This altered the microbiome, consisting of increased *Verrucomicrobia*, *Bacteroidaceae*, *Bacteroides*, and *Akkermansia*, and resulted in increased serum cholesterol, triglycerides, high-density lipoproteins, and low-density lipoproteins (LDL), along with Kyoto Encyclopedia of Genes and Genomes (KEGG) pathways associated with the Janus kinase-stat (JAK-stat) pathway, cytokine receptor interactions, and chemokine and Toll-like receptor (TLR) signaling [20]. Furthermore, long-term antibiotic users have been identified as having an increased risk for ACVD due to increased gut dysbiosis. Kappel and colleagues (2020) examined the metabolic signatures of human subjects with atherosclerosis. They found that patients had decreased serum tryptophan and guanidinobutanoate while having increased long-chain fatty acids and monohydroxy fatty acids. When they then compared ApoE^−/−^ mice with antibiotic treatment, they found a similar metabolic phenotype linked to decreased α-diversity and increased atherosclerotic development compared to non-antibiotic ApoE^−/−^ control mice [21]. Although the gut microbiota varies among individuals, the stable presence of core gut bacteria such as *Firmicutes* and *Bifidobacteriaceae* seems to be required for normal gut function. Overall, these studies indicate that several bacteria may influence the development of ACVD via multiple mechanisms.

## 3. The Immune System and ACVD

Although scientists are actively researching the connections between ACVD, the host immune system, and the trillions of gut bacteria, a connecting link has not yet been fully discovered [22]. In atherosclerosis, an initial insult to the endothelial lining leads to endothelial activation and structural alteration, resulting in plasma lipids, macrophages, and other leukocytes being recruited to the arterial wall [23]. Gut microbiota drive changes in the body’s inflammatory state, thus altering atherogenesis. In healthy individuals, the gut bacteria and their metabolites work together to optimally respond to threats via the innate and adaptive immune responses [22,24]. Importantly, butyrate binds to GPR109a (a G protein-coupled receptor) on macrophages and DCs, decreasing the production of IL-6 and elevating the expression of the anti-inflammatory cytokine IL-10 [25]. This results in the development of T-regulatory (T_reg_) cells while suppressing the proliferation of pro-inflammatory Th17 cells [26]. Butyrate has also been shown to regulate T_reg_ cells via forkhead box P3 (Foxp3) differentiation, which is essential for the suppression of several inflammatory responses [25,27]. Thus, one of the roles of butyrate in the immune response is enhancing anti-inflammatory processes and regulating the T_reg_ cell population to protect against systemic inflammation.

In ACVD, a large number of pro-inflammatory cytokines, including Interferon gamma (IFN-γ), IL-12, Transforming growth factor beta (TGF-β), and tumor necrosis factor-alpha (TNF-α) are evident in atherosclerotic plaques [28,29,30]. A greater ratio of pro-inflammatory to anti-inflammatory markers within atherosclerotic plaques may indicate accelerated plaque development and repressed inflammation resolution [31]. Several factors aid the switch between the pro- and anti-inflammatory phenotypes, including the transcription factor peroxisome proliferator-activated receptor-gamma (PPAR-γ), a nuclear receptor mainly expressed in intestinal epithelial cells, which may be crucial for regulating atherogenesis. Chemokines and their receptors are expressed extensively throughout the body but are especially prominent in cells crucial to atherosclerosis development, including endothelial cells, smooth myocytes, and leukocytes. There, they promote atherosclerosis by selectively recruiting leukocytes and by activating G protein-coupled receptors (GPCRs), triggering chemotaxis [32].

Aside from their involvement in PPAR signaling, commensal bacteria also inhibit phagocyte migration, hence decreasing T and B cell activation. Commensal bacteria also initiate goblet cell differentiation, leading to the expansion of the protective mucosal layers. Contrarily, pathogenic bacteria activate DCs, which then trigger a specific T cell response including the differentiation of naive T cells into Th1 and Th17 cells [33,34]. Lipopolysaccharide (LPS), found in the cell membrane of Gram-negative bacteria, contains several moieties including a core oligosaccharide, a lipid A moiety, and an O-antigen polysaccharide [35]. The lipid A moiety is recognized by TLRs, specifically the TLR4/MD-2 complex, which, upon stimulation, activates the innate immune response [36]. LPS serves as a representative pathogen-associated molecular pattern (PAMP) that can be used to identify specific bacterial pathogen invasions. Mucosal surface cells sense the presence of PAMPs through pattern recognition receptors (PRRs), triggering the innate immune response [36]. The other function of the polysaccharide moiety of LPS is to protect the pathogenic bacteria, both by preventing complement attacks and by camouflaging the bacteria with standard host carbohydrate residues [36]. This ability enables these bacteria to evade the host’s innate immune system, allowing for greater pathogenicity. However, an overabundance of SCFA-producing bacteria radically reduces the proliferation of Gram-negative bacteria and therefore decreases LPS levels [37]. By influencing these various immune system responses, the gut microbiota can heavily impact the course of atherosclerotic development and perpetuation.

Many intracellular cascades are involved in ACVD pathophysiology, including JAK signal transducer, mitogen-activated protein kinase (MAPK), activator of transcription (STAT), and protein kinase B (Akt). Akt has an essential role in the proliferation and migration of endothelial cells, vascular penetration control, and angiogenesis within the vascular wall [38]. Knocking out Akt2, which is essential for metabolism, results in reduced glucose tolerance and increased insulin resistance, both hallmarks of ACVD [39,40,41]. JAK/STAT signaling is involved in a wide variety of essential cell processes but is particularly key for activating the cellular stress response. In cardiovascular disease, elevation of the STAT gene leads to foam cell formation, atherosclerotic plaque development, and increased expression of pro-inflammatory signals [42]. MAPKs also play a diverse role in cardiovascular physiology and are abundant in atherosclerotic lesions [43,44]. When researchers compared untreated groups to SB203580-treated groups—a known inhibitor of p38 MAPK—they found amplified gut inflammation, gut dysbiosis, and severe acute pancreatitis in the untreated groups. When analyzing the SB203580-treated group, the researchers found increased microbial diversity among the Bacteroidetes, Firmicutes, and Proteobacteria phyla [45]. This may be due to MAPKs’ known role in initiating the pro-inflammatory response, releasing cytokines, growth factors, and oxidative stress. Together, these pathways shape the course of atherosclerotic lesion development both in the vasculature and in the gut.

## 4. The TMA/TMAO Pathway

The gut microbiota anaerobically breaks down fatty foods high in cholesterol, yielding the precursors phosphatidylcholine (PC) and L-carnitine [46]. Choline-TMA lyases are the main enzymes related to TMA formation from choline, produced from facultative anaerobic microbes. These enzymes cut the carbon–nitrogen (C–N) bond of PC, freeing the waste product TMA [47,48].

**Figure 1:**
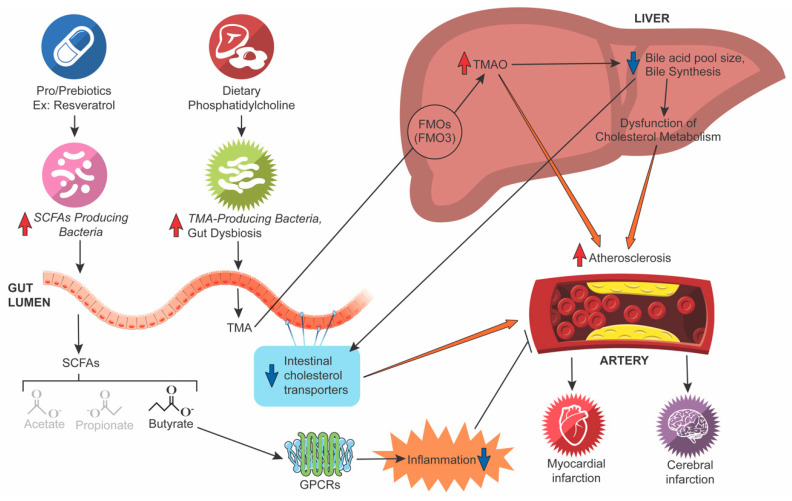
**Mechanisms by which microbiota participate in atherosclerosis.** The dietary nutrients choline and carnitine are selectively converted into TMA by TMA-producing and metabolizing enzymes from anaerobic gut microbiota. TMA is transferred to the liver via portal circulation and oxidized by the hepatic flavin monooxygenase 3 (FMO3), resulting in the production of TMA–N-oxide (TMAO). Increasing TMAO has been demonstrated to decrease the bile acid pool size and inhibit bile acid synthesis, impacting cholesterol metabolism via altered intestinal cholesterol transport, specifically reverse cholesterol transport (RCT). TMAO has also been associated with enhanced macrophage cholesterol accumulation and atherosclerosis development. Supplementation with pro/prebiotics, such as resveratrol, can increase butyrate-producing bacteria. With the increase in butyrate-producing bacteria, TMA-producing bacteria become less prominent as they are outcompeted for resources. This results in increased levels of butyrate, which binds to G protein-coupled receptors (GPCRs), leading to decreased intestinal inflammation and an overall decreased risk of atherosclerosis, myocardial infarction, and cerebral infarction.

L-carnitine is converted to γ-butyrobetaine (γbb) which is converted into TMA and TMAO. For example, select facultative anaerobic bacteria, such as *Actinobacteria* and *Proteobacteria*, utilize γbb and directly convert L-carnitine to TMA through dependent carnitine monooxygenase (CntA) [46,47,49]. TMA is then delivered to the liver, where a family of host hepatic enzymes, specifically the flavin monooxygenase 3 (FMO3), oxidizes TMA to form TMAO [50,51] (see Figure 1).

Wang et al. (2011) showed an association between the risk of atherosclerosis in humans and the plasma concentrations of choline, TMAO, and betaine but also demonstrated their proatherogenic abilities in mice [7]. For example, supplementing mouse diets with betaine, choline, or TMAO stimulated an increased expression of several atherosclerosis-linked macrophage scavenger receptors, but only choline and TMAO supplementation resulted in increased atherosclerosis. Additional germ-free mouse studies confirmed dietary choline and gut flora play a critical role in TMAO formation, accumulation of cholesterol in macrophages, and foam cell formation. Establishing a greater understanding of the connection between the gut microbiota metabolism of dietary PC and ACVD development would improve the ability to create novel diagnostic tests and treatment options for ACVD [7]. In 2014, Tang and colleagues observed higher fasting plasma TMAO levels in heart failure patients compared to age- and gender-matched control groups. They also noted an especially robust negative prognosis associated with raised circulating TMAO levels among a group of stable heart failure patients that were incremental to classic risk factors, cardiorenal indicators, and signs of systemic inflammation [50]. Hoyles et al. conducted shotgun sequencing with molecular phenomics on fecal samples taken from morbidly obese women, with or without liver steatosis, and found steatosis was linked to decreased microbial gene richness, an increased presence of *Proteobacteria*, *Actinobacteria*, and *Verrucomicrobia*, and a decreased presence of *Firmicutes* and *Euryarchaeota*. When they performed a fecal microbial transplant from these patients to mice, they were able to replicate the increase in hepatic triglycerides, plasma valine concentration, increase in circulating valine, leucine, and isoleucine (and their dysregulated metabolism), and increase in circulating TMAO seen in the human subjects [52]. Thus, this study provides evidence that gut microbiota-dependent TMAO generation may contribute to more than just the development and progression of ACVD (see Table S1—Supplementary Materials).

Increased TMAO levels can be used as a prognostic tool for evaluating CVD risk [53,54,55]. Elevated TMAO is independently linked to the frequency of CVD and risks for MI, stroke, and death [56]. Likewise, increased plasma concentrations of betaine, choline, and carnitine are independently linked to an increased risk of MI, stroke, and death. However, their predictive values are largely limited to individuals with concurrently raised TMAO levels [51]. One study demonstrated that TMAO levels are related to enrichment in the *Bacteroides* and *Prevotella* genera and showed that dietary TMAO supplementation encouraged a decline in total cholesterol absorption in mice. They also observed that TMAO precursors, choline and carnitine, along with TMAO repress reverse cholesterol transport (RCT) in vivo through intestinal microbiota-dependent mechanisms [55]. Results from recent studies in high-fat diet-fed mice indicate dietary TMAO supplementation might block hepatic insulin signaling, intensify diminished glucose tolerance, and augment inflammation in adipose tissue due to the upregulated expression of genes involved in these pathways [57]. Thus, TMAO can be directly linked to several comorbidities of ACVD.

To demonstrate the therapeutic potential of manipulating TMAO levels, Chen and colleagues (2016) found that resveratrol, a natural phytoalexin with prebiotic characteristics, diminished TMAO-induced atherosclerosis in ApoE knockout mice. Simultaneously, resveratrol remodeled the gut microbiota, resulting in higher levels of the genera *Bifidobacterium* and *Lactobacillus.* This led to the inhibition of TMA production, and an overall reduction in TMAO levels. As a result, bile salt hydrolase (BSH) enzymatic activity increased, thus enhancing BA deconjugation and increasing fecal excretion [58]. When broad-spectrum antibiotics were administered to wild-type mice, there was a near total suppression of TMAO levels due to intestinal flora suppression [7]. However, one month after stopping antibiotic treatment, TMAO levels were once again detectable in plasma. Other mouse studies used a mix of several antibiotics and were able to inhibit dietary choline-accelerated atherosclerosis, suppress TMAO plasma concentrations, and inhibit macrophage foam cell development [9]. Unfortunately, despite this promising suppression of TMA-producing microbiota, chronic antibiotic use is not a feasible treatment option because it can result in resistant bacterial strains and repopulation (see Table S1).

## 5. SCFAs and Inflammation

Certain commensal intestinal bacteria, including *Anaerostipes butyraticus*, *Faecalibacterium prausnitzii*, and *Roseburia intestinalis*, digest complex carbohydrates and produce SCFAs, which supply an energy source for colonocytes and modulate the host immune system [59,60,61]. SCFAs are strongly related to reducing intestinal inflammation, preserving barrier stability, and protecting against pathogen invasion. The most prominent SCFAs are acetate, butyrate, and propionate [62]. Their primary function is to regulate the immune response, largely via the generation of T_reg_ cells and suppression of histone deacetylases (HDACs), both of which play crucial roles in atherosclerosis [27,63]. This SCFA-driven inhibition of HDACs suppresses the inflammatory response and consequently disrupts DC development [26,64]. Administration of SCFAs to peripheral blood mononuclear cells resulted in downregulated NF-κB activation and decreased pro-inflammatory cytokine production [65]. Thus, SCFAs’ ability to function as HDAC inhibitors may help regulate the inflammatory pathways in ACVD.

Additionally, research has implicated SCFAs as regulators of liver cholesterologenesis [66]. There is a marked difference in the hepatic metabolism of germ-free and bacteria-colonized mice, potentially due to differences in the hepatic generation of SCFAs [67]. In the liver of bacteria-colonized mice, scientists observed evidence of increased triglyceride synthesis including elevated levels of stored triglycerides and triglyceride transporter production. This was accompanied by a decrease in fasting-induced adipose factor expression within the small intestine. One such factor, angiopoietin-like 4 (ANGPTL4), normally inhibits the activities of lipoprotein lipase (LPL), including its mediation of adipose cell triglyceride uptake [68]. ANGPTL4 has been identified as a downstream target of PPARs, agonists of which are extensively employed as treatments for CVD and T2DM [69,70]. PPAR-γ serves as the master regulator of adipocyte formation, whereas PPAR-α is primarily involved in hepatic fatty acid oxidation [69]. Of note, ANGPTL4 can be regulated by the gut microbiota [53]. When germ-free mice and ANGPTL4-deficient mice were fed a high-fat diet, they gained a substantially greater body weight and adipose tissue compared to high-fat diet-fed colonized mouse controls. Thus, ANGPTL4 directly modulates the gut microbiome’s ability to regulate mouse adiposity [71,72]. Ingesting SCFA-producing bacteria may be a viable method for promoting the hepatic influx of SCFAs, yielding increased ANGPTL4 regulation, and consequently reducing the risk of developing ACVD.

Due to their antioxidative and pro-apoptotic properties, SCFAs can attenuate the oxidative and pro-inflammatory characteristics of ACVD. Aguilar and colleagues (2014) provided butyrate supplementation to several models of atherosclerosis: a human endothelial cell line and an ApoE knockout mouse model [73]. Butyrate impeded the development of atherosclerosis by increasing plaque stability and by diminishing the adhesion and migration of pro-inflammatory macrophages. Butyrate was also associated with reduced CD36 expression in macrophages and endothelial cells, lowered activation of NF-κB, and decreased release of pro-inflammatory cytokines [73]. Together, these studies solidify SCFAs’ role as anti-inflammatory atheroprotective agents. Administering propionate, another SCFA, in mouse drinking water clearly attenuated hypertension, vascular inflammation and atherosclerosis, and cardiac damage in two different hypertensive cardiovascular damage mouse models [47]. This effect largely depended on propionate’s ability to regulate immune homeostasis, particularly with regard to T_reg_ function. Marques et al. sought to unravel which improvements in cardiovascular functions could be attributed to high fiber intake vs. acetate [74]. Acetate, the most abundant SCFA, was effective at normalizing cardiac and renal hypertrophy and function, improving hypertension, and reducing the left ventricular wall thickness in a hypertensive C57Bl/6 mouse model [74]. It also led to decreases in body weight, which is an important risk factor for the development of atherosclerosis. Though acetate alone did not lead to changes in IL-1 signaling, fiber and acetate administration was associated with decreases in the transcription factor Egr1, the reduction in which is commonly associated with protection from inflammation [74]. Thus, oral supplementation with propionate could improve cardiovascular indices in human patients. 

## 6. Bile Acid (BA) Metabolism

BAs are steroid acids produced by the liver and come in two forms. Primary BAs are directly synthesized from hepatic cholesterol and are conjugated with glycine, resulting in the production of chenodeoxycholic acid and cholic acid. These primary BAs ensure fats and vitamins are soluble and readily absorbed. Once the primary BAs pass into the duodenum, they are then reabsorbed into the distal ileum where they undergo deconjugation facilitated by the resident gut bacteria to form secondary BAs. BAs are involved in nutrient absorption and foreign substance disposal but can also act as signaling molecules [75]. They bind to and activate the nuclear hormone receptor farnesoid X receptor (FXR) and G protein-coupled bile acid receptor (TGR5), which are strongly associated with impairing and maintaining glucose homeostasis in the body [76,77].

Activation of the TGR5s induces type 2 deiodinase activity in brown adipocytes, leading to enhanced energy expenditure. TGR5 is also involved in increasing glucagon-like peptide 1 (GLP-1) secretion from enteroendocrine cells. Together, this results in improved glucose tolerance [78,79]. One study found that using a semisynthetic BA (INT-777) to activate macrophage TGR5s induced adenosine 3’,5’-cyclic monophosphate (cAMP) signaling, subsequently inhibited NF-κB, and resulted in inhibited pro-inflammatory cytokine production. Miyazaki-Anzai et al. supported the importance of TGR5 and provided evidence for FXRs as anti-atherosclerotic targets. When they administered INT-767, a dual agonist of FXR and TGR5, they found it blocked classic atherosclerotic formation and decreased concentrations of the typical aortic cytokines and chemokines in low-density lipoprotein receptor knockout (LDLR^−/−^) mice with a single deficiency of either FXR or TGR5. The anti-inflammatory and anti-atherogenic effects of INT-767 were completely obstructed when LDLR^−/−^ mice were deficient in both FXR and TGR5. However, the ability to lower lipid levels was completely repressed when the mice were singularly FXR deficient, but not when singularly deficient in TGR5 [80]. This suggests that, while FXR and TGR5 are both important for reducing atherosclerosis and inflammation, FXR’s role in lowering lipids is not critical for INT-767’s ability to reduce atherosclerotic lesion size. Hu and colleagues (2018) used a high-fat diet rat model to demonstrate INT-767’s anti-inflammatory and anti-atherogenic effects. INT-767 exerted its anti-inflammatory effect by suppressing the TNF-α and NF-κB signaling pathways, significantly alleviating the liver damage caused by the high-fat diet [81]. This indicates that BA receptor activation is highly involved in modulating immune function and in preventing ACVD.

## 7. Other Factors

**Figure 2:**
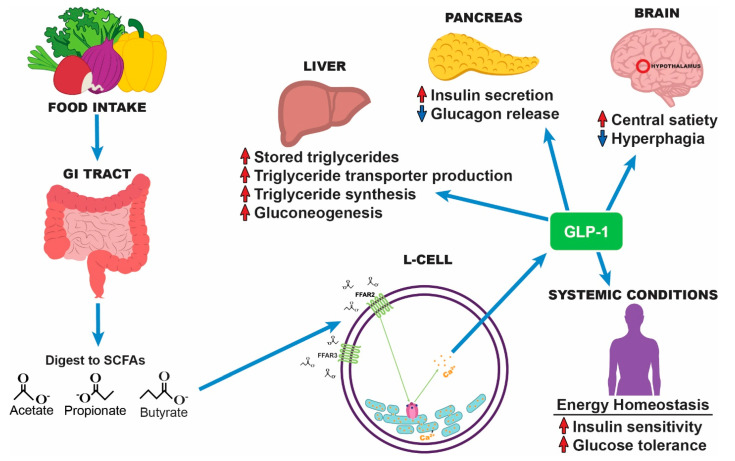
**SCFAs bring about system-wide improvements in ACVD risk factors via GLP-1.** Following food intake, dietary fibers are digested by gut microbiota into SCFAs including acetate, propionate, and butyrate. These SCFAs activate intestinal L cells, leading to the release of GLP-1 both independently of and in conjunction with FFAR2 and FFAR3 activation. GLP-1 modulates a variety of organ functions including the liver, pancreas, and brain. It also systemically improves insulin sensitivity and glucose tolerance. This ability to improve various cardiovascular and metabolic risk factors for ACVD implicates GLP-1 and its synthetic mimetics as a viable treatment option for atherosclerosis.

The absolute association between the gut microbiome and ACVD development is still unknown. A variety of demographic characteristics such as age, sex, and ethnicity may alter gut microbiota, cholesterol levels, and even a person’s diet. However, chronic low-grade inflammation and T2DM have been strongly implicated as risk factors in the development of ACVD [6]. One hypothesis posits that increased insulin resistance can result in compensatory hyperinsulinemia, which is one of the metabolic irregularities believed to underlie the pathophysiology of metabolic syndrome. Metabolic syndrome, in turn, is a precursor to ACVD [82]. Additionally, the excessive buildup of visceral fat, as in morbid obesity, is correlated with insulin resistance. This condition leads to raised production of pro-inflammatory cytokines and macrophage infiltration, fostering chronic low-grade inflammation and diminished immune–insulin interactions [83]. However, SCFAs can mitigate the T2DM-associated biological disruptions as they have demonstrated functional interactions with other endocrine hormones, such as leptin, ghrelin, peptide YY, and GLP-1. SCFAs play an essential role in T2DM by binding to GPCRs and generating a variety of downstream consequences, including insulin resistance. These metabolites are critical for lowering inflammation, defending against pathogen infiltration, and preserving intestinal barrier integrity by binding to GPCRs and inhibiting the activity of histone deacetylases (HDACs) [24,48]. Specifically, SCFAs have demonstrably enhanced GLP-1 secretion. GLP-1 is an incretin hormone produced by the gut that is essential for glucose homeostasis. Following a meal, dietary fibers are metabolized into SCFAs, which then activate intestinal L cells. These L cells release GLP-1, which modulates glucagon release, hepatic gluconeogenesis, insulin secretion, insulin sensitivity, and central satiety [84]. Therapies such as GLP-1, GLP-1 mimetics, and SCFAs have been widely used to maintain body weight in obese individuals and to manage blood glucose levels in T2DM patients. Intravenous GLP-1 administered to T2DM patients reduces hyperglycemia and promotes insulin secretion via ion channel regulation. GLP-1 receptor agonists act as incretin mimetics and are linked with glucose-lowering effects, leading to a reduction in hemoglobin A1c (HbA1c) and consequently aiding in weight loss. Administration of an SCFA cocktail significantly elevated colonic GLP-1 secretion in rat and human L cell lines [85]. Moreover, administering butyrate-induced bacteria yielded therapeutic effects including protection against weight gain, increased GLP-1 secretion, and decreased insulin resistance [86]. How SCFAs enhance GLP-1 secretion is currently undetermined. It is believed that the free fatty acid receptors (FFAR)2 (GPR43) and FFAR3 (GPR41) promote GLP-1 secretion [85,87] (see Figure 2). Additionally, activation of OLFR78 (olfactory receptor 78) has been shown to raise blood pressure, while activation of FFAR3 has been shown to reduce blood pressure [88]. Other studies showed that the SCFA-induced increase in GLP-1 secretion occurred independently of FFAR2 (GPR43) and FFAR3 (GPR41) expression in an animal model [89]. Continuing to explore the mechanisms by which GLP-1 alleviates diabetes-associated inflammation is critical for identifying potential therapeutic avenues and therefore reducing ACVD incidence.

Accumulation and oxidative modification of LDL cause focal fibroinflammatory degeneration of the arterial intima, a characteristic of atherosclerosis [38,90]. Focal fibroinflammatory degeneration is modulated by adaptive immune responses against modified self-antigens in atherosclerotic plaques [29]. The harmony between protective immunity and inducing disease relies on how antigen-presenting cells (APCs) present antigens to T cells [91]. Interferon-γ, the pro-inflammatory cytokine, induces histocompatibility complex (MHCII) molecules. These molecules are activated on several cell types, including endothelial cells, macrophages, and smooth muscle cells, and are a precondition for T cell and APC activation [92,93]. The activation of adaptive immune responses through MHCII is related to the development of ACVD. Wigren and colleagues (2019) observed that the plasma level of CD4+ T cells and Th1 and Th2 cytokines, and immunoglobulin levels (especially IgG and IgM), decreased, whereas CD8+ T cells were increased in ApoE^−/−^MHCII^−/−^ mice administered a high-fat diet. The reduced inflammatory cytokine levels in ApoE^−/−^MHCII^−/−^ mouse plasma indicated reduced systemic inflammation. In spite of this, ApoE^−/−^MHCII^−/−^ mice had significantly more atherosclerosis due to losing regulatory T cells [94]. In a human study, CD4+ cell and CD8+T cell counts were also alternated in patients with cardiovascular risk factors. The activation of CD4+T cells in response to oxidized LDL antigen triggers the formation and promotes the propagation of arterial thrombosis, resulting in MI, while CD8+T cells induce the rupture of a developed atheromatous plaque by their cytotoxic nature [95].

## 8. Conclusions

The most common underlying cause of cardiovascular disease is the atherosclerotic process. This process is also related to risk factors including chronic inflammation, diabetes, and obesity. The alteration in the gut microbial composition leads to imbalances in the consequent levels of metabolites. Repeatedly, TMAO has been implicated in the progression of ACVD. Repressing TMAO-producing bacteria has been shown to reduce the onset of dietary-choline-enhanced atherosclerosis in a mouse model. Higher fasting plasma TMAO levels have also been observed in heart failure patients compared to healthy controls. Additionally, TMAO precursors, choline and carnitine, have been linked to reduced RCT. Meanwhile, elevated TMAO itself may aggravate many of the symptoms seen in consumers of a high-fat Western diet, including insulin resistance. Similarly, suppression of SCFA production can also lead to severe issues such as intestinal inflammation, decreased gut barrier integrity, reduced immune tolerance, and decreased protection against foreign pathogens. This can also cause dysregulation of liver cholesterol synthesis, macrophage polarization towards a pro-inflammatory phenotype, and reduced atherosclerotic plaque stability. Finally, when BA production is diminished, a variety of metabolic actions are interrupted, such as the absorption of fats and vitamins, foreign substance disposal, glucose and lipid metabolism, regulation of the TNF-α and NF-κB signaling pathways, and the maintenance of glucose homeostasis. Together, the effects of these reductions in gut metabolites strongly suggest the gut microbiota is paramount in the development of ACVD. Still, when looking into these various bacterial strains and the functions of their metabolic products, it is impossible to deny their strong connections to other diseases. Obesity, diabetes, and metabolic syndrome are associated with gut dysbiosis and can also put an individual at a higher risk for developing ACVD. Potential treatment strategies for ACVD may help to mitigate the onset of these other risk factors.

Over the last decade, there has been continuous research regarding therapeutic interventions for ACVD. For example, statins are drugs prescribed for primary and secondary prevention of ACVD due to their lipid-lowering activity, and independent anti-inflammatory effects, such as decreased C-reactive protein levels [96]. Researchers have also found that TNF blockade reduces the risk of ACVD events in RA patients by inhibiting the expression of certain pro-inflammatory chemokines and cytokines, specifically TNF-α, but not IL-6 [97]. Although inhibiting the IL-6 receptor seemed to have a therapeutic effect in RA patients, there was a concerning increase in lipid levels, a potential risk factor for ACVD. Alternatively, active immunization or antibody infusion has been shown to alter the balance of pro-inflammatory and anti-inflammatory T cells, and to expand T_reg_ cells in animal models, an immune process which is critical for atherosclerotic lesion development [98]. With the increased availability of gene therapy, scientists have begun to investigate specific RNA targets expressed within the liver as a potential avenue for treating ACVD, with varying degrees of success [99,100]. Non-immune therapies include ingesting SCFA-producing probiotics or administering antibiotics, both of which have been shown to provide some degree of gut remodeling. This could lead to inhibition of the gut bacterial TMA production, increase BSH enzyme activity, and increase the production of SCFA-producing bacteria. These treatment strategies may not be effective under prolonged use, and, as in the case of antibiotics, long-term use could produce unwanted negative side effects. Therefore, it may be better to selectively target particular bacterial strains for upregulation or downregulation within the gut microbiota. Administering resveratrol as a prebiotic was successful in remodeling the gut microbiota and saw no negative side effects. Specific probiotic metabolites such as SCFAs, BAs, or their derivatives have also been promising when used in cellular and animal models, but verification of their efficacy in humans is still necessary.

As the prevalence of risk factors for ACVD continues to rise, there remains a critical need for further investigation into atherosclerosis treatments. This is especially important due to ACVD’s role as the most common cause of death worldwide. Carefully evaluating the gut microbiota–immune interactions will enable the identification of novel predictors of ACVD and the expansion of available treatment strategies, thus closing the gap between ACVD onset and the availability of effective treatments.

## Supplementary Materials

The following supporting information can be downloaded at: https://www.mdpi.com/article/10.3390/microorganisms10010108/s1, Table S1: The role of gut microbiota in TMA and TMAO pathways.

## Abbreviations

APCs	Antigen-presenting cells
ApoE^−/−^	Apolipoprotein E knockout
ACVD	Atherosclerotic cardiovascular disease
ANGPTL4	Angiopoietin-like 4
cAMP	Adenosine 3′,5′-cyclic monophosphate
BAs	Bile acids
FXR	Bile acid-activated nuclear hormone receptor farnesoid X receptor
CVD	Cardiovascular diseases
FMOs	Flavin monooxygenases
Foxp3	Forkhead box protein P3
GLP-1	Glucagon-like peptide 1
GPCRs	G protein-coupled receptors
TGR5	G protein-coupled bile acid receptor
HbA1c	Hemoglobin A1c
LPS	Lipopolysaccharide
LDL	Low-density lipoprotein
NF-κB	Nuclear factor-κB
OLFR78	Olfactory receptor 78
PRRs	Pattern recognition receptors
PYY	Peptide YY
PPARs	Peroxisome proliferator-activated receptors
SCFAs	Short-chain fatty acids
TMA	Trimethylamine
TMAO	Trimethylamine-N-oxide
TNF-α	Tumor necrosis factor-alpha
Treg	T-regulatory
Th17	T helper 17 cell
TLRs	Toll-like receptors
T2DM	Type 2 diabetes
