# Supplementary material for: Gut Dysbiosis and Immune System in Atherosclerotic Cardiovascular Disease (ACVD)
Source: Microorganisms. 2022 Jan 5;10(1):108. doi: 10.3390/microorganisms10010108 (PMC8780459; doi:10.3390/microorganisms10010108)
Supplement: Supplementary file 1 [file microorganisms-10-00108-s001.zip › microorganisms-1545563-supplementary.pdf]

Table S1: The role of gut microbiota in TMA and TMAO pathways.
↓ indicates decreasing whereas ↑ indicates increasing.

Species	Alternation of Gut Microbiota Taxonomy	Proposed mechanisms	Reference
Human	Stroke patients compared to controls:<br>↑ *Lactobacillus ruminus*<br><br>Ischemic stroke independently associated with: ↑*Atopobium* cluster<br>↑ *L. ruminus*<br>↓ *L. sakei*<br><br>T2D associated with:<br>↓ *C. coccoides*	↑ *L. ruminis* → ↑ inflammation in stroke patients (↑ IL-6)<br><br>Ischemic stroke → ↓ acetic acid + ↑ valeric acid<br>↓ acetic acid → ↑ HbA1c + LDL cholesterol<br>↑ valeric acid → ↑ CRP + leukocyte counts<br><br>↓ *C. coccoides* → ↑ HbA1c + ↑ LDL cholesterol + ↑ CRP + IL-6	1) Yamashiro et al., 2017, PLOS One
Human and Mice	*Desulfovibrio desulfuricans* degrades choline → TMA via choline-specific TMA lyase cutC/D<br><br>*Acinetobacter baumannii:* degrades carnitine → TMA via carnitine-specific TMA lyase cntA/B	<u>In humans:</u><br>↑ circulating carnitine, choline, or betaine (dietary precursors to TMA/TMAO) → ↑ risk of myocardial infarction, stroke, or death independent of traditional risk factors, but only when ↑ TMAO levels<br>↑ plasma choline, ↑ betaine, + ↑ TMAO → phosphatidylcholine (PC) metabolism<br><br><u>In mouse models:</u><br>Dietary exposure to TMA or precursors → ↓ Reverse cholesterol transport (RCT), alteration in cholesterol + sterol metabolic pathways<br>↑ Dietary L-carnitine → ↑ atherosclerosis pathogenesis when gut microbiota intact<br><br>So, ↓ direct TMA precursor/substrate consumption or modifying gut microbial composition → ↓ ability to produce TMA	2) Tang & Hazen, 2014, The Journal of Clinical Investigation
Human and Mice	None	<u>In humans:</u><br>↑ Plasma levels of choline, TMAO and betaine → ↑ atherosclerosis risk<br><br><u>In atherosclerosis prone (C57BL/6J $Apoe^{-/-}$) mice:</u><br>↑ dietary choline, TMAO, + betaine → ↑ ACVD lesion area + ↑ CD36 + SR-A1 in macrophages<br>↑ dietary choline and TMAO → minimal change in plasma choline, ↑ plasma TMAO levels<br>↑ plasma levels of TMAO → ↑aortic lesion size<br>↑ dietary choline → ↑ lipid-laden macrophage development<br>↑ hepatic FMO3 expression → ↑ atherosclerotic lesion formation, ↓ HDL cholesterol, + ↑ plasma TMAO<br><br><u>In atherosclerosis prone mice given antibiotics (ABX):</u><br>Admin of d9-PC or d9-choline ≠ ↑ plasma TMAO, but restored when reintroduced to normal mice<br>↑ dietary choline ≠↑ macrophage foam cell formation or ↑ atherosclerosis or ↑ CD36 expression<br>Choline supplementation promotes macrophage foam cell formation in a gut-flora-dependent fashion<br><br><u>In germ free mice:</u><br>Admin of d9-PC or d9-choline ≠ ↑ plasma TMAO but restored when reintroduced to normal mice<br><br><u>Identified pathway:</u><br>dietary PC/choline → gut-flora-formed TMA → hepatic-FMO-formed TMAO	3) Wang et al., 2011, Nature
Human and Mice	<u>In humans:</u><br>↑ *Bacteroides* → ↑ plasma TMAO	<u>In humans:</u><br>↑ fasting plasma [carnitine] → ↑ risk of coronary artery disease, peripheral artery disease, and overall	4) Koeth et al., 2013, Nature Medicine

	↑ *Prevotella* → ↑↑ plasma TMAO<br><br>Vegans (compared to omnivores) + ↓ TMAO → ↓ *Clostridiaceae,* ↓ *Peptostreptococcaceae incertae sedis,* ↓ *Peptostreptococcaceae,* ↓ *Clostridium,* + ↑ *Lachnospira*<br><br><u>In mice:</u><br>↑ dietary carnitine + ↑ TMA → ↑ *Prevotella* + ↑ Prevotellaceae *Unclassified*<br>↑ dietary carnitine + ↑ TMAO → ↑ *Anaeroplasma* + ↓ Porphyromonadaceae	CVD<br>↑↑ fasting plasma [carnitine] → ↑ risk of major adverse cardiac events only when no adjustment made for [TMAO]<br><br>Vegetarian and vegan fasting TMAO levels < omnivore fasting TMAO levels<br>Vegetarianism/veganism → ↓ dietary l-carnitine or choline → ↓ capacity for synthesis of TMAO from l-carnitine → ↓ TMAO levels → ↓ CVD<br>Omnivores → ↑ dietary l-carnitine → ↑ capacity for synthesis of TMAO from l-carnitine → ↑ atherosclerosis<br><br><u>In Apoe –/– mice:</u><br>↑ dietary l-carnitine → ↑plasma carnitine, ↑ production of TMA + TMAO, + ↑↑ disease burden<br>↑ dietary l-carnitine or ↑ dietary choline → ↓ RCT compared to normal chow-fed controls<br>TMAO-containing diet → 35% ↓ in RCT compared to normal chow-fed controls<br>Dietary TMAO supplement → ↓ mRNA hepatic levels of key bile acid synthetic enzymes Cyp7a1 and Cyp27a1 +<br>↓ bile acid transporter expression (Oatp1, Oatp4, Mrp2, Ntcp) in the liver, but not the gut, + ↓ total bile acid pool size<br><br>ABX + ↑ dietary l-carnitine → ↓ plasma TMA and TMAO levels + complete ↓ dietary l-carnitine–dependent increase in atherosclerosis, but ↑ plasma carnitine concentrations<br>ABX + ↑ dietary l-carnitine or ↑ dietary choline ≠ ↓ RCT compared to normal chow-fed controls<br><br>So, microbial composition changes→ changes in TMAO synthetic capacity → altered sterol metabolism<br>Also, TMAO, rather than carnitine = primary driver of the correlation between carnitine and CVD risk	
Human	<u>In humans with ACVD:</u><br>↑ *Streptococcus,* ↑ *Escherichia,* ↓ *Bacteroides,*<br>↓ *Prevotella,* ↓ *Alistipes shahii,*<br>↑ *Enterobacteriaceae* (*Escherichia coli, Klebsiella* spp., and *Enterobacter aerogenes*),<br>↑ *Streptococcus* spp., ↑ *Lactobacillus salivarius,* ↑ *Solobacterium moorei*, ↑ *Atopobium parvulum* ↑ *Ruminococcus gnavus,*+ ↑ *Eggerthella lenta*<br><br>↓ butyrate-producing bacteria (*Roseburia intestinalis* and *Faecalibacterium cf. prausnitzii)*<br><br>↑ an unclassified *Erysipelotrichaceae* bacterium, *C. nexile*, + *S. anginosus* encode CutC → ↑ TMA synthetic capacity<br><br>↑ E. aerogenes and Klebsiella pneumoniae encode the TMA lyase YeaW/X → ↑ TMA synthetic capacity	<u>In ACVD patients:</u><br>Metagenomic linkage groups differentially enriched in people with versus without ACVD<br><br>Gut microbiome showed ↑ potential for transport of simple sugars (phosphotransferase systems) and amino acids, but ↓ potential for biosynthesis of most vitamins, ↓ potential for the synthesis of tetrahydrofolate, changed potential for homocysteine metabolism, ↓ potential for metabolizing glycans (e.g. glycosaminoglycans), ↑ potential for metabolism of glycerolipids and degradation of fatty acids, ↓ potential for synthesis of anti-inflammatory butyrate,<br>↓ module involved in propionate synthesis, ↑ Gut microbial enzymes involved in formation of TMA<br><br>↑ Enterobacteriaceae in ACVD → ↑ gene module for synthesis of the O-antigen of LPS<br>↓ gram-negative genus *Bacteroides* → ↓ lipid A synthesis module<br><br>Alterations in gut microbial functional modules in ACVD and other disease included phosphotransferase transport systems, amino acid transporters, vitamin metabolism, and LPS biosynthesis.<br>Cardiometabolomic disease ↔ ↓ fermentative + ↑ inflammation of the gut microbiome	5) Jie et al., 2017, Nature Communications

Mice	n/a	In transintestinal cholesterol excretion mouse models, FMO3 gene ↓ → regulator of RCT<br>FMO3 antisense oligonucleotide (ASO) treatment → no change in overall health of mice, ↓ hepatic cholesteryl ester levels, ↓ hepatic FMO3 mRNA and protein expression compared to nontargeting control ASO → ↑ TMA + ↓ TMAO<br>FMO3 ASO treatment → ↓↓ intestinal cholesterol absorption, + ↑ fecal neutral sterol loss in low-cholesterol diet mice + ↓ cholesterol absorption, ↓ VLDL cholesterol levels, ↑ LDL cholesterol levels, but no change in fecal sterol loss in high-cholesterol diet mice<br><br>So, knockdown (KD) of FMO3 → reorganization of cholesterol balance in a diet-specific manner, suggesting a link between FMO3 and cholesterol and BA metabolism.<br><br>FMO3 KD → ↓ expression of oxysterol synthetic enzymes Cyp27a1 + Cyp46a1 → ↓ availability of endogenous oxysterol ligands in liver → ↑ SREBP2-driven transcription and ↓ LXR signaling<br><br>FMO3 KD → ↓ total plasma cholesterol levels, ↑ basal + ↑ LXR agonist-stimulated macrophage RCT, but ↓ biliary cholesterol levels + ↓ intestinal cholesterol absorption<br><br>FMO3 KD →↓ LXR activation → ↑ activation of c-Src, ↑ hepatic ER stress (↑ ATF3, CHOP) + inflammation (↑ infiltration of macrophages into the liver, ↑ macrophage-derived proinflammatory cytokine + chemokine expression)<br>FMO3 KD + LXR agonists → ↓ FMO3 ASO-driven hepatic inflammation, c-Src activation, + ER stress<br><br>Even though chronic TMAO is proatherogenic, it is most likely not involved in the mechanism by which FMO3 inhibitors reorganize cholesterol balance and inflammation of the liver.	6) Warrier et al., 2105, Cell Reports
Human, Mice, and Rat	<u>In humans:</u><br>↓ *Bacteroidetes*:*Firmicutes* ratio → obesity<br>ACVD → ↑ *Collinsella*<br>Healthy controls → ↑ *Roseburia* + ↑ *Eubacterium*<br><br>↑ *Tenericutes* + ↑ *Christensenellaceae* associated with ↓ BMI, ↓ triglyceride (TG), + ↑ HDL levels → ↑ acetate (SCFA)<br><br>↑*Peptococcaceae,* ↑*Prevotella*, + ↓ *Faecalibacterium prausnitzii* → ↑ TMAO<br><br><u>In hypertensive animals:</u><br>Observed ↓ microbial diversity and ↓ *Bacteroidetes*:*Firmicutes* ratio observed.<br><br><u>In mice:</u><br>ABX-induced dysbiosis → non-pathogenic *Salmonella enterica* transport to the mesenteric lymph nodes → T cell response and IgA production	<u>In humans:</u><br>Bacterial dysbiosis → overproduction of nitrogenous compounds → disruption of intestinal epithelial tight junctions → translocation of gut bacterial DNA and uremic toxins into circulation: e.g. atherosclerotic plaques include bacterial DNA (mostly *Proteobacteria*)<br><br>SCFAs = signaling molecules → bind to G-protein coupled receptors GPR41 and GPR43<br>SCFA bind to GPR43 → regulation of the inflammatory response: both GPR43-deficient mice and germ-free mice → ↑ production of inflammatory mediators<br>SCFAs → inhibit NF-κB → ↓ inflammatory cytokine production<br><br>Phosphatidylcholine and other TMA containing compounds (L-carnitine or choline) → metabolized by gut microbiota TMA lyases → release TMA → TMA metabolized by FMOs → produce TMAO<br><br>Found a dose-dependent association between plasma TMAO levels and platelet aggregation<br><br>In T2DM, ↑ TMAO levels → ↑ risk of adverse cardiovascular events and mortality, independent of glycemic control<br>Fecal microbiota transplant from lean donor to insulin-resistant people with metabolic syndrome → ↑ insulin sensitivity + ↑ butyrate-producing gut bacteria<br>Insulin → ↓ FMO3 expression → ↑ TMAO levels ; Glucagon → ↑ FMO3 expression → ↓ TMAO levels<br><br>↓ SCFAs → ↓ insulin sensitivity and ↓ insulin-mediated fat accumulation<br><br><u>In mice:</u><br>Mice on choline or TMAO supplemented diets → ↑ platelet hyperreactivity and thrombosis risk	7) Ahmadmehrabi & Tang, 2017, Curr Opin Cardiol

		compared to germ-free mice on the same diet<br><br>KD of ↑ FMO3 → ↑ insulin tolerance, ↓ hypercholesterolemia, and ↓ atherosclerosis<br><br>↑ SCFAs in high-fat diet fed mice without changing food intake or exercise → ↓ body weight + ↑ insulin sensitivity<br><br>GPR41 receptor-deficient mice = systolic hypertensive phenotype, implying SCFA signaling reduces blood pressure<br><br><u>In rats:</u><br>TMAO infusion → ↑ the hypertensive effects of angiotensin II	
Cultured Mouse Cecum Bacteria and Mice	Administration of DMB:<br>↓ the rate of intact *P. mirabilis* conversion of d9-choline → d9-TMA<br>↓ many bacterial taxa positively associated with TMA, TMAO, or aortic lesion area<br>↑ many bacterial taxa negatively associated with TMA, TMAO, or aortic lesion area<br><br>But the effect varied:<br>*D. alaskenesis* showed ↓ inhibition<br><br>DMB = non-lethal inhibitor of *P. mirabilis* → no ↓ cell growth, ↓ TMA lyase activity<br>*Proteus penneri* and *Escherichia fergusonii* = choline TMA lyase activity<br>↑ DMB in cultures of *Proteus penneri* or *Escherichia fergusonii* → ↓ choline utilization → ↓ TMA, but no change in bacterial growth<br><br>Proportions of several taxa = aortic root lesion area and plasma [TMA] + [TMAO]<br>E.g. In male mice, ↑ dietary choline → ↑ *Clostridiaceae* → ↑ plasma [TMA] + [TMAO], + ↑ atherosclerotic lesion area<br>But ↑ dietary choline + DMB admin → ↓ *Clostridiaceae*<br><br>E.g. In female mice,<br>↑ dietary choline → ↑ *Clostridiales* → ↑ plasma [TMA] + [TMAO] + ↑ atherosclerotic lesion area<br>But ↑ dietary choline + DMB admin → ↓ *Clostridiales*<br><br>↑ *Lachnospiraceae* + ↑ *Ruminococcus* → ↑ [TMA], [TMAO], + ↑ plaque area	A choline analog, 3,3-dimethyl-1-butanol (DMB) → ↓ microbial choline TMA lyase activity<br><br>DMB → ↓ some but not all microbial TMA lyases and inhibits TMA formation from multiple substrates in physiological polymicrobial cultures<br><br>Wild type (WT) *E. coli* BL21 strain ≠ carnitine TMA lyase activity<br>Transformed *E.coli* cells (w/cntA or cntB from *A. baumannii*) ≠carnitine TMA lyase activity individually<br>*E. coli* cells (w/cntA + cntB) → expected acquired enzymatic activity → cleaved d9-carnitine → d9-TMA<br><br>DMB = non-lethal inhibitor of TMA production by microbes<br>DMB → ↓ plasma TMAO levels in vivo<br><br>DMB → ↓ choline-diet-enhanced macrophage foam cell formation and ↓ atherosclerosis<br>DMB ≠ effect on choline uptake by the microbes → DMB does not block choline uptake into the cells<br><br>Proves concept: ↓ microbial TMA lyase activity → ↓ microbial TMA production → potential therapeutic approach for the prevention or treatment of atherosclerosis<br><br>DMB admin, despite no significant effects on circulating cholesterol, choline, and other pro-atherogenic risk factors → ↓ choline-diet-dependent accumulation of both foam cell formation + aortic root atherosclerotic plaque development<br><br>Some DMB-induced change in microbial composition → degree of selective pressure is occurring with exposure to the agent → possibility for the development of resistance	8) Wang et al., 2015, Cell Press

	↑ *Clostridiales* → ↑ plasma TMA levels<br><br>↑ S24-7 (*Bacterioidetes*) → ↓ [TMA], ↓ trend [TMAO], + ↓ ACVD plaque area		
Human	↑ *Klebsiella, Streptococcus, Haemophilus,* + *Granulicatella* in more severe CAD<br><br>The bacterial co-abundance groups (CAGs) → age, inflammatory markers (hs-CRP and IL-18), blood lipids and dietary fiber intake<br><br>↑ CAD → ↑ CAG17 (*Veillonella, Haemophilus,* + *Klebsiella*) = pathogens<br>CAG4 (Faecalibacterium and Roseburia) = 10 serum modules → important in maintenance of normal coronary artery homeostasis<br><br>↑ CAD development → ↓ CAGs containing OTUs from butyric acid-producing Lachnospiaceae and Ruminococcaceae<br><br>Severe CAD → ↑ CAGs containing OTUs from Ruminococcaceae) → ↑ *Clostridium*	Identified 29 metabolite modules associated with coronary artery disease (CAD) phenotypes<br>Over the course of CAD, the gut microbiome composition changes dramatically, as does the metabolic phenotype<br>Compared to healthy controls, CAD patients = disruptions in glucose and lipid metabolism, + ↑ inflammation<br><br>↑ CAG17 → ↑ innate immune response<br><br>↑ CAD-associated metabolites → ↑ main risk factors of CAD, but ↓ cholesterol<br>↑ phosphatidylethanolamine, PC, phosphatidylserine, and sphingolipid metabolites → ↓ AS severity and myocardial markers<br>↑ Taurine + hypotaurine metabolic module → ↓ CAD severity<br>↑ Aromatic compounds like bacterially produced benzenoids → disrupted CAD development<br><br>Some bacteria may affect atherosclerosis by modulating host metabolic pathways like taurine, sphingolipid and ceramide, and benzene metabolism	9) Liu et al., 2019, Microbiome
Mice	RSV → gut microbiota remodeling:<br>↑ *Lactobacillus* + ↑ *Bifidobacterium*	Resveratrol (RSV) admin → ↓ TMAO-induced atherosclerosis in ApoE –/– mice<br>RSV → gut microbiota remodeling → ↑ bile salt hydrolase activity → ↑ BA deconjugation and fecal excretion in C57BL/6J and ApoE –/– mice → ↓ BA in the ilea, ↓ gut-liver FXR-FGF15 axis, ↑ CYP7A1 expression, and ↑ liver BA synthesis<br><br>FXR antagonist = RSV effect on FGF15 and CYP7A1 expression<br>FXR agonist → ↓ RSV effect on FGF15 and CYP7A1 expression<br><br>ABX → ↓ RSV inhibition of TMAO-driven atherosclerosis<br><br>So RSV → ↓ TMA producing bacteria → ↓ [TMAO], ↑ BA synthesis, mediated by FXR-FGF15 axis	10) Chen et al, 2016, mBio
Human, Mice, and Cultured Human Hepato-cytes	↑ *Proteobacteria, Actinobacteria,* + *Verrucomicrobia* → ↑ liver steatosis<br>↑ *Firmicutes* + *Euryarchaeota* → ↓ liver steatosis<br>↓ species diversity = ↑ liver steatosis	<u>In Humans:</u><br>LPS + peptidoglycan biosynthesis = liver steatosis<br>124 urine metabolite signals + 80 (plasma) →liver steatosis<br>Most liver steatosis-associated metabolites = ↓ microbial gene richness<br>e.g. ↑ BCAAs (plasma + urine) → liver steatosis<br>e.g. ↓ choline and phosphocholine (plasma) = liver steatosis<br>↑ choline excretion = liver steatosis<br>↓ microbial gene richness (steatosis patients) → ↓ plasma PAA<br><br>↑ microbial gene richness (non-steatotic patients) → ↑ urinary phenylacetylglutamine, plasma acetate plasma acetate, + plasma TMAO<br>↑ TMAO (not TMA) = ↓ steatosis by UPLC-MS/MS<br>Hepatic genes = core immune response to clearance of viral and bacterial infections, alcoholism + insulin resistance	11) Hoyles et al, 2018, Nature Medicine

		↑ microbial gene richness = ↓ KEGG pathways (proteasome, phagosome, insulin resistance, glucagon signaling and non-specific responses to microbial (Gram-negative, viral) infections)<br>Hepatic steatosis → ↑ LPL (lipoprotein lipase)<br>Hepatic steatosis → ↓ short/branched chain acyl-CoA dehydrogenase + insulin receptor<br><br><u>In mice:</u><br>Fecal microbial communities from donors with hepatic steatosis transplanted into recipient mice<br>Steatosis mice = ↑ hepatic triglycerides, Fabp4 expression, + plasma valine concentration<br>Donor microbiota composition → mouse phenome<br>Steatosis-associated microbiota → hepatic triglycerides, circulating BCAAs + TMAO<br>steatosis mice + PAA → ↑ hepatic triglycerides, ↓ excreted isoleucine<br><br><u>In primary human hepatocytes:</u><br>PAA → triglyceride accumulation molecular mechanisms, ↑ expression of lipid metabolism genes (*LPL* and *FASN*), ↑ *INSR* expression, ↓ *GLUT2* expression, ↓ AKT phosphorylation, ↑ short/branched chain acyl-CoA dehydrogenase expression, ↑ utilization of BCAA from medium<br><br>Metabolic phenotype (↑ BCAAs, AAAs and microbial metabolites) → hepatic steatosis and low microbial gene richness	
Human and Mice	<u>In humans:</u><br>atherosclerosis = ↓ members of *Bacteroidetes* + *Clostridia*<br><br><u>In mice:</u><br>ABX → ↑ cecal content weight, ↓ cecal content DNA (bacterial count), ↓ α-diversity → ↑ atherosclerotic lesion size<br><br>ABX → ↑*Brucellaceae* (normal diet), ↑*Streptococcacae* (Western diet)<br><br>↑*Clostridia* (*Lachnospiraceae*, *Ruminococcaceae*) + *Bacteroidetes* (*Porphyromonadaceae*, *Rikenellaceae*) = ↑Tryptophan metabolism + secondary bile acid metabolism	<u>In humans:</u><br>Human subjects w/carotid atherosclerosis→ ↓ serum tryptophan, ↑ long-chain fatty acids, ↑ monohydroxy fatty acids, ↓ guanidinobutanoate<br><br><u>In mice:</u><br>ApoE-/- + ABX → ↑ atherosclerotic lesion size<br>ApoE-/- + Western diet → ↑ atherosclerotic development at aortic root level<br>Western Diet → ↓ α-diversity→ ↓ metabolic diversity (serum) → ↑ atherosclerosis + lesion size<br>Tryptophan supplementation alone ≠ atherosclerosis<br>ABX + tryptophan supplementation → ↓ aortic lesion size<br><br>ABX → tryptophan metabolism, secondary bile acid metabolism, pyrimidine metabolism, cytidine containing, polyunsaturated fatty acids and food component/plant<br>Lesion size → ↓ tryptophan metabolism + secondary bile acid metabolism, ↓ fatty acid + dihydroxy fatty acids, ↑ lipid metabolism<br><br>ABX → ↓ TMAO by antibiotics → no contribution of TMAO to the phenotype	12) Kappel et al, 2020, Molecular Metabolism
Human	Both atherosclerosis patients = ↓*Bacteroides xylanisolvens, Odoribacter splanchnicus, Eubacterium eligens, Roseburia inulinivorans,* + *Roseburia intestinalis*<br><br>Swedish patients = ↓*B xylanisolvens* + *E eligens, Bifidobacterium adolescentis* + *Collinsella aerofaciens* vs controls<br>Swedish controls = ↑*Bacteroides*	Metagenomics of stool samples from atherosclerosis patients in Sweden (n=25) and China (n=385)<br><br>↑*Clostridium* sp L2-50, *E eligens*, *Coprococcus comes*, *Lachnospiraceae* bacterium 1 1 57FAA, + *Lachnospiraceae* bacterium 5 1 63FAA → ↓CRP<br>↑*Anaerostipes hadrus, Turicibacter sanguinis, Akkermansiamuciniphila, Clostridium celatum, Bacteroides finegoldii,* + *Haemophilus parainfluenzae* → ↑HDL<br>↑*Bacteroides finegoldii, B xylanisolvens,* + *Haemophilus parainfluenzae* → ↓WBC and triglycerides<br><br>Patients = ↑Pathways L-arginine biosynthesis IV (archaebacteria), I (via L-ornithine), + II (acetyl cycle)	13) Liu et al, 2020, The FASEB Journal

	caccae, B dorei, B fecis, B finegoldii, + B xylanisolvens<br><br>Chinese patients = ↑*Firmicutes, Bacteroides fragilis, Streptococcus salivarius, Clostridium nexile, Ruminococcus gnavus, Ruminococcus torques, Escherichia coli, Klebsiella pneumoniae*, + *Akkermansia muciniphila*<br>Chinese controls = ↑ *Faecalibacterium prausnitzii, Prevotella copri,* + *Bacteroides uniformis*<br><br>Both controls = ↑*Roseburia inulinivorans* + *Roseburia intestinalis*<br>Chinese controls = ↑*Bacteroides* + ↓*Firmicutes* vs Swedish controls	Controls = ↑Pathways: Starch degradation V (PWY-6737), CDP-diacylglycerol biosynthesis I/II (PWY-5667/PWY0-1319), L-lysine biosynthesis III/VI (PWY-2942/PWY-5097), glycolysis III from glucose (ANAGLYCOLYSIS-PWY), queuosine biosynthesis (PWY-6700), folate transformations II (PWY-3841), N10-formyl-tetrahydrofolate biosynthesis (1CMET2-PWY)<br><br>*B xylanisolvens, E eligens,* + *R inulinivorans* = potential probiotics/target for atherosclerosis	
Mice	Phyla: ApoE-/- → ↑*Verrucomicrobia*<br><br>Family: ApoE-/- → ↑*Ruminococcaceae* + *Bacteroidaceae*<br>ApoE-/- → ↓*Rikenellaceae*<br><br>Genus: Apoe-/- → ↑*Bacteroides* + *Akkermansia*<br><br>↑*Verrucomicrobia*, *Bacteroidaceae*, *Bacteroides*, + *Akkermansia* → ↑ serum total cholesterol, triglyceride (TG), HDL, + LDL<br>↑*Ruminococcaceae* → ↑ HDL<br>↑*Rikenellaceae* → ↓ TG and LDL<br><br>↑*Akkermansia* + *Verrucomicrobia* → ↓CXCL5, FGF2, E-Selectin, GITR, CXCL11, + TIMP2<br>↑*Bacteroides* + *Bacteroidaceae* → ↓CXCL5 + CXCL11<br>↑*Bacteroides* + *Bacteroidaceae* → ↑CCL22<br>↑*Ruminococcaceae* → ↓CXCL5 + CXCL11<br>↑*Rikenellaceae* → ↑FGF2 + GITR	α-diversity of ApoE-/- + high-fat diet (HFD) = α-diversity of WT mice + HFD<br>Over time, fecal bacteria composition in ApoE-/- mice ≠ WT mice as atherosclerosis developed<br><br>ApoE-/- = ↑ IFN-γ, IL-6, + MCP-1<br><br>ApoE-/- = ↑ signaling pathways: fluid shear stress, atherosclerosis, Jak-STAT, + cytokine-cytokine receptor interaction<br>ApoE-/- = ↑ major immune pathways: chemokine, Toll-like receptor signaling	14) Liu et al, 2020, Microbial Pathogenesis
